# Use of Anti-angiogenic Drugs Potentially Associated With an Increase on Serum AST, LDH, CK, and CK-MB Activities in Patients With Cancer: A Retrospective Study

**DOI:** 10.3389/fcvm.2021.755191

**Published:** 2021-12-02

**Authors:** Qi Zheng, Hanzhou Wang, Wei Hou, Ying Zhang

**Affiliations:** ^1^Department of Pneumology, Guang'anmen Hospital, China Academy of Chinese Medical Sciences, Beijing, China; ^2^Department of Oncology, Guang'anmen Hospital, China Academy of Chinese Medical Sciences, Beijing, China

**Keywords:** anti-angiogenic drugs, cancer, AST, LDH, CK, CK-MB

## Abstract

**Background:** There is a large amount of evidence that anti-angiogenic drugs are effective safe. However, few studies have evaluated the specific effects of anti-angiogenic drugs on myocardial enzyme injury biomarkers: aspartate aminotransferase (AST), lactic dehydrogenase (LDH), creatine kinase (CK) and creatine kinase isoenzyme (CK-MB). The purpose of our study was to determine whether anti-angiogenic drugs serum AST, LDH, CK, and CK-MB activities of cancer patients treated with anti-angiogenic drugs.

**Methods:** This study retrospectively analyzed 81 cancer patients. Patients who had used anti-angiogenic drugs were selected. Serum AST, LDH, CK, and CK-MB activities were measured before and after treatment with anti-angiogenic drugs for 3 weeks.

**Results:** A total of 16 cancer types were analyzed. The distribution of the cancer types in the patients was mainly concentrated in lung, gastric, and colorectal cancers. The anti-angiogenic treatment markedly increased AST, LDH, CK, and CK-MB activities by 32.51, 7.29, 31.25, and 55.56%, respectively in serum.

**Conclusions:** Our findings suggest that patients, who had used anti-angiogenic drugs were likely to have elevated AST, LDH, and CK, indicators of myocardial muscle injury. Use of anti-angiogenic drugs should not be assumed to be completely safe and without any cardiovascular risks.

## Introduction

Anti-angiogenic treatment is an effective and targeted therapy strategy that can be used to control and kill tumors (1). Although chemotherapeutics can kill tumor cells, the remaining tumor cells can still survive and continue to grow due to the support of peripheral blood vessels. Meanwhile, abnormal tumor blood vessels reduce the delivery of drugs into tumor tissues, which ultimately leads to limited efficacy of anti-cell proliferation therapy. Therefore, the treatment for cancer should not only be directed against tumor cells, but also against the tumor microenvironment, in particularly tumor angiogenesis (2).

Vascular endothelial growth factor (VEGF) is the master effector of the angiogenic response in cancers (3). Anti-angiogenic drugs can be used to specifically bind to VEGF to prevent it from interacting with receptors, which play a critical role in tumor blood vessels. Further, this limits exposure to oxygen and other nutrients required for tumor cell growth, thereby weakening the ability of tumor growth and metastasis. Anti-angiogenic agents targeting the VEGF and HIF-α pathways include monoclonal antibodies to VEGF (4), such as bevacizumab and resumumab, small-molecule tyrosine kinase inhibitors (TKIs), such as anlotinib, sorafenib, and sunitinib, and VEGF receptor (VEGFR)2 inhibitors, such as regorafenib and ramucirumab (5). These compounds can lead to a reduction in the tumor blood supply and growth of the tumor blood vessels. Unfortunately, cardiovascular toxicity is a potential limitation associated with the long-term use of anti-angiogenic agents in cancer and requires further study to assess the value of anti-angiogenic treatment.

Aspartate aminotransferase (AST) is a pyridoxal-5′-phosphate-dependent enzyme that is widely distributed in heart, liver, skeletal muscle, kidney and brain. It plays a key role in the metabolism of amino acids, synthesis of purine/pyrimidine bases, urea and protein synthesis, and gluconeogenesis (6). Lactic dehydrogenase (LDH) is a type of enzyme, which plays an important role in making body's energy. It can be found in almost all the body's tissues, including those in the blood, heart, kidneys, brain, and lungs. LDH is released from damaged tissues, and can serve as a biomarker for damaged heart tissue. Creatine kinase (CK) is a guanidino-kinase that catalyzes the reversible phosphorylation of creatine to phosphocreatine, and is primarily distributed in bone and myocardium. The plasma activity of creatine kinase isoenzyme (CK-MB), one of the isoenzymes of CK, is generally used to evaluate acute coronary syndrome. The detection of serum CK isozymes, especially serum CK-MB, is helpful for judging the degree of myocardial injury. Comprehensively, monitoring serum AST, LDH, CK and CK-MB activities for cardiac biomarkers can be valuable for assessing patient status (7, 8).

Unfortunately, few studies have focused on the measuring changes in serum AST, LDH, CK and CK-MB activities before and after anti-angiogenic treatment for cancer. In this study, we conducted a retrospective investigation focused on measuring the changes in serum AST, LDH, CK and CK-MB in serum on cancer patients receiving anti- angiogenic targeted therapy. The results suggested that in serum AST, LDH, CK and CK-MB activities of patients who had used anti-angiogenic drugs were likely to have elevated.

## Materials and Methods

### Patients

This was an observational, retrospective study that obtained informed consent from all subjects, and this research was approved by the Ethics Committee of Guang'anmen hospital, China Academy of Chinese Medical Sciences with code number 2020-073-KT. The study followed the ethical principles of the Declaration of Helsinki 1964.

From Jan 2014 to Dec 2020, cancer 81 patients treated with apatinib, anlotinib, regorafenib, bevacizumab, sorafenib, or sunitinib at the oncology department, Guang'anmen hospital, China Academy of Chinese Medical Sciences were retrospectively recruited for this study. Patients with active infection, systemic corticosteroid treatment within 1 year, or hematological malignancy were excluded since these conditions might affect the hematological laboratory markers. Meanwhile, 81 gender and age matched healthy control were enrolled from physical examination center, Guang'anmen hospital, China Academy of Chinese Medical Sciences.

### Data Collection

The following variables were extracted from the medical records of the patients: AST, LDH, CK and CK-MB results, age, gender, histological diagnosis, and the choice of anti-angiogenic drugs (such as apatinib, anlotinib, regorafenib, bevacizumab, sorafenib, or sunitinib), history of prior heart disease, pharmacohistory of cardiovascular drugs, smoking and drinking history, and effect of chemotherapeutic drugs on cardiotoxicity. Additionally, routine complete blood counts and coagulograms (including the AST, LDH, CK and CK-MB activities) were carried out before and after first 1 cycle of therapy The first effective evaluation was proceeded after 21 days of treatment. All the examining were detected in the laboratory department, Guang'anmen hospital, China Academy of Chinese Medical Sciences, by using full-automatic chemistry analyzer (AU5800 series, Beckman Coulter).

### Statistical Analysis

Statistical analyses were performed using GraphPad Prism8 (GraphPad Software, San Diego, CA, USA) and SPSS statistical software version 24.0 (SPSS Inc., Chicago, IL, USA). Serum AST, LDH, CK and CK-MB activities after first one cycle of treatment were compared to that with no treatment were analyzed. Considering the predictor variables, such as age, gender, histological diagnosis, and the choice of anti-angiogenic drugs, history of prior heart disease, pharmacohistory of cardiovascular drugs, smoking and drinking history, and effect of chemotherapeutic drugs on cardiotoxicity, the statistics were done by mixed linear modeling. All data were non-normally distributed, that reported by the median, interquartile range, and min-max. The differences between tumor patient group and matched healthy control group were compared with Mann-Whitney U test. A two-sided *P* < 0.05 was deemed as statistically significant.

## Results

### Patient Characteristics

A total of 81 patients were treated by anti-angiogenic drugs during the study period. Table 1 presents the detailed patient characteristics. There were 41 (50.6%, 95% CI: 0.397 ~ 0.615) males and 40 (49.4%, 95% CI: 0.385 ~ 0.603) females in the total cohort, with a median age of 63 years (Quartiles 25–75%, 56–70). Among the patients, there were 42.0% (34/81, 0.312 ~ 0.527) have prior heart disease history, 35.8% (29/81, 0.254 ~ 0.462) have cardiovascular drugs use history. Meanwhile, a total of 34.6% (28/81, 0.242 ~ 0.449) have smoking history and 23.4% (19/81, 0.142 ~ 0.327) have drinking history. All the patients have been treated with different chemotherapy, nevertheless, there were 3/81 patients were affected by chemotherapeutic drugs on cardiotoxicity.

**Table 1:** Patients' characteristics.

**Variables**	**N**	**%**	**95% CI**	
**Age**				
Median	63			
Quartiles 25–75%	56–70			
**Gender**				
Male	41	50.6	0.397 ~ 0.615	
Female	40	49.4	0.385 ~ 0.603	
**Prior heart disease history**				
Yes	34	42.0	0.312 ~ 0.527	
No	47	58.0	0.473 ~ 0.688	
**Cardiovascular drugs use history**				
Yes	29	35.8	0.254 ~ 0.462	
No	52	64.2	0.538 ~ 0.746	
**Smoking history**				
Yes	28	34.6	0.242 ~ 0.449	
No	53	65.4	0.551 ~ 0.758	
**Drinking history**				
Yes	19	23.4	0.142 ~ 0.327	
No	62	76.5	0.673 ~ 0.858	
**Effect of chemotherapeutic drugs on cardiotoxicity**				
Yes	3	3.7	−0.004 ~ 0.078	
No	78	96.3	0.922 ~ 1.004	
**Cancer type**				**tumor stage**
Lung cancer	23	28.4	0.186 ~ 0.382	IV
Gastric cancer	13	16.1	0.081 ~ 0.240	III~IV
Colorectal cancer	15	18.5	0.101 ~ 0.270	IV
Ovarian cancer	5	6.2	0.009 ~ 0.114	IV
Liver cancer	5	6.2	0.009 ~ 0.114	IV
Renal cancer	4	4.9	0.002 ~ 0.097	IV
Metrocarcinoma	3	3.7	−0.004 ~ 0.078	IV
Esophagus cancer	2	2.5	−0.009 ~ 0.058	IV
Pancreatic cancer	2	2.5	−0.009 ~ 0.058	IV
Urethral carcinoma	2	2.5	−0.009 ~ 0.058	IV
Osteocarcinoma	2	2.5	−0.009 ~ 0.058	IV
Breast cancer	1	1.2	−0.012 ~ 0.036	IV
Cholangiocarcinoma	1	1.2	−0.012 ~ 0.036	IV
Thyroid cancer	1	1.2	−0.012 ~ 0.036	IV
Duodenal cancer	1	1.2	−0.012 ~ 0.036	IV
Thymoma	1	1.2	−0.012 ~ 0.036	IV
**Anti-angiogenic therapy**				
Apatinib	27	33.3	0.231 ~ 0.436	
Anlotinib	25	30.9	0.208 ~ 0.409	
Regorafenib	12	14.8	0.071 ~ 0.226	
Bevacizumab	10	12.3	0.052 ~ 0.195	
Sorafenib	6	7.4	0.017 ~ 0.131	
Sunitinib	1	1.2	−0.012 ~ 0.036	

A total of 28.4% (23/81, 95% CI: 0.186 ~ 0.382) patients had lung cancer, 16.1% (13/ 81, 95% CI: 0.081 ~ 0.240) had gastric cancer, 18.5% (15/81, 95% CI: 0.101 ~ 0.270) had colorectal cancer, 6.2% (5/81, 95% CI: 0.009 ~ 0.114) had ovarian cancer, and 6.2% (5/81, 95% CI: 0.009 ~ 0.114) had liver cancer. A small number of renal cancer (4.9%, 4/81), metrocarcinoma (3.7%, 3/81), esophagus cancer (2.5%, 2/81), pancreatic cancer (2.5%, 2/81), urethral carcinoma (2.5%, 2/81), osteocarcinoma (2.5%, 2/81), breast cancer (1.2%, 1/81), cholangiocarcinoma (1.2%, 1/81), thyroid cancer (1.2%, 1/81), duodenal cancer (1.2%, 1/81), and thymoma (1.2%, 1/81) cases were included too. Except one patient is III gastric cancer, all other cancer patients were at staged IV depending on the TNM (Tumor, lymph Node, distant Metastasis). In addition, 27 of 81 patients (33.3%, 95% CI: 0.231 ~ 0.436) were treated with apatinib, 25 of 81 patients (30.9%, 95% CI: 0.208 ~ 0.409) were treated with anlotinib, 12 of 81 patients (14.8%, 95% CI: 0.071 ~ 0.226) were treated with regorafenib, 10 of 81 patients (12.3%, 95% CI: 0.052 ~ 0.195) were treated with bevacizumab, six patients were treated with sorafenib, and one patient (12.3%) were treated with sunitinib (Table 1).

### Evaluation of Serum AST and LDH Activities in Patients and Health Control

**Figure 1:**
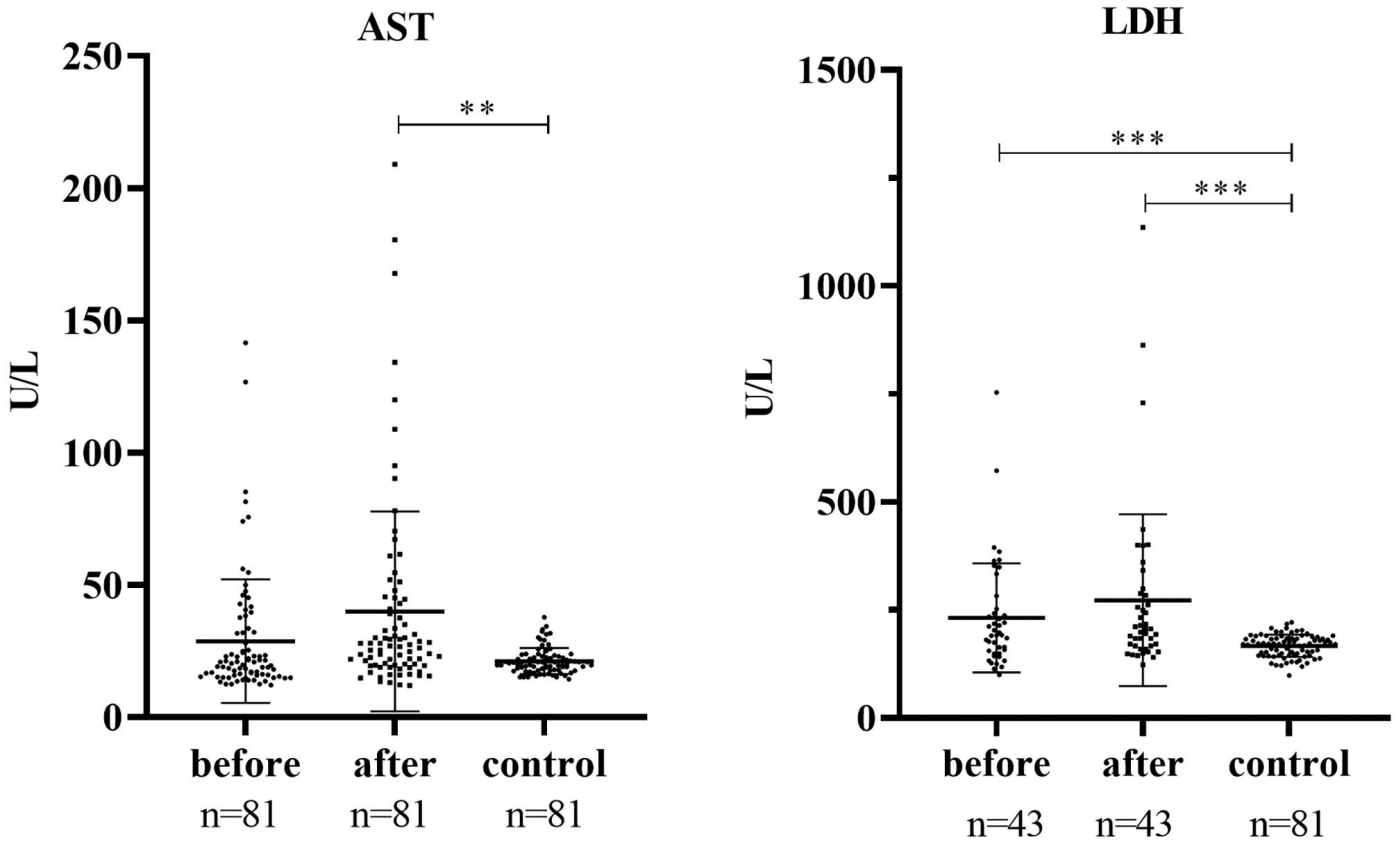
Serum AST and LDH activities in patients and health control. ***P < 0.001, **P < 0.001.

Table 2 displays the detailed characteristics of serum AST, LDH, CK, and CK-MB activities in patients and health control. Health control was matched in serum AST, LDH. Serum AST and LDH were markedly increased in patients after treatment compared with health control (*p* < 0.01, *p* < 0.001). In addition, it was significantly increased in patients before treatment compared with health control in serum LDH (*p* < 0.001), (Figure 1).

**Table 2:** The detailed characteristics of serum AST, LDH, CK and CK-MB activities in patients and health control.

	**Group**	**Number**	**Median**	**Interquartile range**	**Min**	**Mix**
AST	Before treatment	81	18.40	10.20	12.10	126.70
	After treatment	81	26.70	24.00	12.00	209.10
	Health control	81	19.90	6.20	14.30	37.80
LDH	Before treatment	43	192.00	103.00	100.00	753.00
	After treatment	43	206.00	122.00	123.00	1,135.00
	Health control	81	176.00	98.00	119.00	217.00
CK	Before treatment	43	32.00	31.00	12.00	144.00
	After treatment	43	42.00	40.00	10.00	244.00
CK-MB	Before treatment	47	9.00	217.52	0.48	218.00
	After treatment	47	13.00	20.00	0.35	252.00

*In our laboratory, 90–50 U/L is adopted as the cut-off value for normal AST; <247 U/L is adopted as the cut-off value for normal LDH; <171 U/L is adopted as the cut-off value for normal CK; <25 U/L is used as the cut-off value for normal CK-MB*.

### The Effects of Predictor Variables on Serum AST, LDH, CK, and CK-MB Activities in Patients

Afterwards, considering the predictor variables, such as age, gender, histological diagnosis, and the choice of anti-angiogenic drugs, history of prior heart disease, pharmacohistory of cardiovascular drugs, smoking and drinking history, and effect of chemotherapeutic drugs on cardiotoxicity, the statistics were done by mixed linear modeling. As Tables 3–6 showed, the influence of age, gender, cancer type, the choice of anti-angiogenic drugs, drinking history, and effect of chemotherapeutic drugs on cardiotoxicity were eliminated in this study.

**Table 3:** Type III test of fixed effects of AST.

**Source**	**Numerator df**	**Denominator df**	**F**	**Sig**.
Age	1	53.000	0.593	0.445
Gender	1	53	0.672	0.416
Cancer type	15	53	1.679	0.084
Choice of anti-angiogenic drugs	5	53.000	0.550	0.738
Before/after treatment	1	80	10.287	0.002
History of prior heart disease	1	53.000	0.140	0.710
History of cardiovascular drugs	1	53.000	0.984	0.326
Smoking history	1	53	0.000	0.999
Drinking history	1	53.000	0.042	0.838
Effect of chemotherapeutic drugs on cardiotoxicity	1	53.000	0.029	0.866

**Table 4:** Type III test of fixed effects of LDH.

**Source**	**Numerator df**	**Denominator df**	**F**	**Sig**.
Age	1	21.002	0.147	0.705
Gender	1	21.000	0.591	0.451
Cancer type	10	21.006	1.353	0.268
Choice of anti-angiogenic drugs	4	21.006	1.225	0.330
Before/after treatment	1	42.066	5.672	0.022
History of prior heart disease	0	–	–	–
History of cardiovascular drugs	0	–	–	–
Smoking history	1	21.000	3.814	0.064
Drinking history	1	21.004	0.348	0.561
Effect of chemotherapeutic drugs on cardiotoxicity	1	21.000	1.421	0.247

**Table 5:** Type III test of fixed effects of CK.

**Source**	**Numerator df**	**Denominator df**	**F**	**Sig**.
Age	1	21.000	0.461	0.505
Gender	1	21.000	2.961	0.100
Cancer type	9	21.000	1.608	0.177
Choice of anti-angiogenic drugs	4	21.000	0.897	0.483
Before/after treatment	1	42.000	4.946	0.032
History of prior heart disease	1	21.000	4.299	0.051
History of cardiovascular drugs	1	21.000	5.540	0.028
Smoking history	1	21.000	4.708	0.042
Drinking history	1	21.000	0.798	0.382
Effect of chemotherapeutic drugs on cardiotoxicity	1	21.000	0.324	0.575

**Table 6:** Type III test of fixed effects of CK-MB.

**Source**	**Numerator df**	**Denominator df**	**F**	**Sig**.
Age	1	24.000	0.340	0.565
Gender	1	24.000	1.520	0.230
Cancer type	10	24.000	2.034	0.075
Choice of anti-angiogenic drugs	4	24.000	2.042	0.120
Before/after treatment	1	46.000	1.621	0.209
History of prior heart disease	1	24.000	9.675	0.005
History of cardiovascular drugs	1	24.000	12.163	0.002
Smoking history	1	24.000	3.065	0.093
Drinking history	1	24.000	0.016	0.899
Effect of chemotherapeutic drugs on cardiotoxicity	1	24.000	0.299	0.590

Exclude the influence of the above factors, the median of AST activities were 18.40 (10.20, 12.10–126.70) before treatment and 26.70 (24.00, 12.00–209.10) after anti-angiogenic treatment, which was markedly decreased (*P* = 0.002). The mean AST activities were 11.26 higher after anti-angiogenic treatment than before, Tables 2, 7. The median of LDH activities were 192.00 (103.00, 100.00–753.00) before treatment and 206 (122.00, 123.00–1,135.00) after anti-angiogenic treatment, which was significantly decreased (*P* = 0.022). The mean LDH activities were 38.58 higher after anti-angiogenic treatment than before, Tables 2, 7.

**Table 7:** Estimates of fixed effect of treatment, cardiovascular drugs history, history of prior heart disease, and smoking history on serum AST, LDH, CK, and CK-MB activities.

**Dependent Variable**	**Parameter**	**Estimate**	**SE**	**df**	**t**	**Sig**.	**95% CI**
AST	treatment=before	−11.26	3.51	80	−3.207	0.002	−18.24~-4.27
	treatment =after	0	0	–	–	–	–
LDH	treatment=before	−38.58	16.20	42	−2.382	0.022	−71.27~-5.89
	treatment=after	0	0	–	–	–	–
CK	treatment=before	−14.35	6.45	42	−2.224	0.032	−27.37~-1.33
	treatment=after	0	0				
	cardiovascular drugs history=yes	−84.48	35.89	21	−2.354	0.028	−159.12~-9.84
	cardiovascular drugs history=no	0	0	–	–	–	–
	smoking history=yes	47.38	21.84	21	2.170	0.042	1.97~92.80
	smoking history=no	0	0	–	–	–	–
CK-MB	history of prior heart disease=yes	129.04	41.49	24	3.111	0.005	43.42~214.67
	history of prior heart disease=no	0	0	–	–	–	–
	cardiovascular drugs history=yes	−149.10	42.75	24	−3.488	0.002	−237.34~-60.86
	cardiovascular drugs history=no	0	0	–	–	–	–

Interestingly, serum CK and CK-MB activities have been affected by anti-angiogenic treatment, history of prior heart disease, pharmacohistory of cardiovascular drugs, and smoking history. The mean serum CK activities was 14.35, significantly increased after anti-angiogenic treatment than before (*P* = 0.032). Patients who had cardiovascular drugs history had 84.48 lower serum CK activities (*P* = 0.028) than those without cardiovascular drugs history. Smoking history is another predictor variables, patients who had smoking history was 47.38 higher than those without (*P* = 0.042), Table 7. Serum CK-MB activities were mainly history of prior heart disease and cardiovascular drugs. Patients who had prior heart disease history was 129.04 higher serum CK-MB activities (*P* = 0.005) than those without prior heart disease history. Conversely, Patients who had cardiovascular drugs history was 149.10 lower serum CK-MB activities (*P* = 0.002) than those without cardiovascular drugs history.

## Discussion

This retrospective study revealed the blood biomarkers, such as AST, LDH and CK were markedly increased with use of anti-angiogenic drugs, indicating that use of anti-angiogenic drugs may be related to an increased risk of myocardial damage. Moreover, serum CK and CK-MB activities have been affected by history of prior heart disease, cardiovascular drugs, and smoking. The determination of myocardial enzymes mainly includes AST, LDH, CK and CK-MB. When the cardiomyocytes have inflammation (myocarditis) or necrosis (myocardial infarction) due to various reasons, the enzymes contained in the cardiomyocytes can enter the blood, and the activity (content) of these enzymes in the blood increases. Elevation of these serum markers in this study did not exceed the normal upper limit, but it may indicate a tendency for long-term use to accumulate toxicity.

AST is one of the most important aminotransferases in the body. It is mainly found in tissue cells such as myocardium, liver, skeletal muscle, kidney, pancreas, spleen, lung, red blood cells, as well as in normal human plasma, bile, cerebrospinal fluid, and saliva. Medium, but it cannot be detected in urine without kidney damage. The content of AST in the myocardium is the most abundant, so it has certain significance for the diagnosis of myocardial infarction. When acute myocardial infarction (AMI) occurs, the serum AST activity generally rises to 4–5 times the upper limit of the reference value. If it reaches 10–15 times the upper limit of the reference value, it is often fatal infarction occurred. However, the rise of AST is later than CK in AMI, and recovers earlier than LDH, diagnostic value of AST for AMI is becoming less and less. Nevertheless, AST is an indispensable evaluation index in the clinical research of oncology drug evaluation. The study determined the safety and effectiveness of anti-angiogenic therapy with sorafenib and bevacizumab in patients with advanced HCC and results found that dose-limiting toxicities included hypertension, AST increase, creatinine increase, etc. (9). Patients receive intravenous ramucirumab (8 mg/kg) every 2 weeks were observed in a phase 3 clinical trial. Hypertension (34 [12%] of 277 patients treated with ramucirumab), increased AST concentration (15 [5%]), thrombocytopenia (13 [5%]), etc. were occurred with grade 3 or greater adverse events (10). LDH is an extremely important enzyme that regulates the conversion of pyruvate to lactic acid in anaerobic glycolysis and play an important role in cancer metabolism (11). Meanwhile, LDH is a useful marker for predicting the efficacy of bevacizumab-containing chemotherapy in patients with metastatic colorectal cancer (12). It is widely present in the cytoplasm and mitochondria of tissue cells such as liver, heart, skeletal muscle, lung, spleen, brain, red blood cells, platelets, etc. LDH is a tetramer composed of two different subunits (LDHA and LDHB), forming 5 isoenzymes with M-type and H-type subunits: H4(LD1), MH3(LD2), M2H(LD3), M3H(LD4), M4(LD5). Different tissues have their characteristic isoenzymes. The ratios of LD isoenzymes in the heart, kidney and red blood cells are similar, with LD1 and LD2 dominating. When the myocardium is damaged, the myocardial cell membrane ruptures, and the mitochondria and cytoplasmic substances leak out into the intercellular fluid and periphral blood. In response to the hypoxic characteristic of the tumor microenvironment, cancer cells generate a large amount of lactate *via* the metabolism of glucose and glutamine (13, 14). High levels of LDHA expression serves as a prognostic indicator in patients with different type of cancers (15). LDH increased production of reactive oxygen species and regulate cell apoptosis and autophagy (16). Thus, the role of LDH in tumor biology is more complex and may as a potential target in the treatment of cancer. Although in this study, the elevation of AST and LDH in patients did not exceed the normal upper limit, there was a significant increase in serum AST and LDH activities, compared with the matched healthy control.

But it is regrettable that the serum CK and CK-MB activities of matched healthy control were not found. All healthy control were from a medical examination at our hospital, serum CK and CK-MB activities are not included in the physical examination at present. Interestingly, serum CK and CK-MB activities have been affected by history of prior heart disease, cardiovascular drugs, and smoking.

CK mainly exists in skeletal muscle and cardiac muscle, and brain tissue. CK is an important energy regulating enzyme in the myocardium. Under the energy provided by ATP, it catalyzes the reversible phosphorylation of ATP and creatine to ADP and phosphocreatine in cellular energy metabolism, which can be transported to the cytoplasm and stored. Serum CK can be increased in various types of progressive muscle atrophy. CK begins to increase 2–4 h after AMI and can reach 10–12 times the upper limit of normal. It has higher specificity than AST and LDH for diagnosing myocardial infarction, but the increase of this enzyme lasts for a short time, and it returns to normal after 2–4 days. There are three isoenzyme formations for CK: CK-MB (mostly in the heart), CK-MM (mostly in the muscle), or CK-BB (mostly in the brain) (17). CK-MB activity has been recognized as a specific and sensitive biomarker of clinical and subclinical myocardial injury (8, 18). CK-MB activities are significantly positively correlated with the extent of myocardial injury, so serum CK-MB can be used as a biomarker for AMI (19). The presence of CK-MB in patients with cancers may cause confusion with AMI. Serial determinations of both CK and LDH are of great help in differential diagnosis 3512170. In addition, a previous study demonstrated that an elevated serum CK-MB in cancer patients may be associated with cardiac insufficiency, severe illness status, and have high mortality (20). Even a slight increase in CK-MB indicated the possibility of myocardial infarction (21). There has been no retrospective report focusing on CK, CK-MB and anti-angiogenic therapy. In the present study, both CK and CK-MB levels were significant elevated after use anti-angiogenic drug. Myocardial ischaemia might be the reason for the slight increase in CK and CK-MB. Furthermore, Some studies have demonstrated that CK-MB-to-total-CK ratio could be clinically utilized as a primary screening tool for cancer (22), which is an easily available indicator. In this study, we found that patients who had prior heart disease history had a higher serum CK-MB activities, while in patients who had cardiovascular drugs history had a lower serum CK and CK-MB activities on the contrary. The reason for the result is likely to be that people with previous cardiovascular disease have damage to heart muscle cells, while the drugs reduce the damage, which need to be further studied.

## Conclusions

Our findings suggest that the serum AST, LDH and CK activities of patients who had used anti-angiogenic drugs were likely to have elevated. History of prior heart disease, cardiovascular drugs, and smoking should be considered in the anti-angiogenic treatment. AST, LDH, CK and CK-MB are indicators of myocardial muscle injury, such as myocarditis or myocardial infarction. Use of anti-angiogenic drugs should not be assumed to be completely safe and without any cardiovascular risks. In addition, attention should also be paid to long-term use to accumulate toxicity. Apparently, the number of cases in patients should be expanded and more detailed research should be done in the future.

## Data Availability Statement

The raw data supporting the conclusions of this article will be made available by the authors, without undue reservation.

## Ethics Statement

This was an observational, retrospective study that obtained informed consent from all subjects, and this research was approved by the Ethics Committee of Guang'anmen Hospital, China Academy of Chinese Medical Sciences with code number 2020-073-KT. Written informed consent for participation was not required for this study in accordance with the national legislation and the institutional requirements.

## Author Contributions

QZ and YZ: conception and design and manuscript writing. YZ and WH: administrative support and manuscript edition. QZ and HW: data extraction and data analysis. All authors contributed to the article and approved the submitted version.

## Funding

This work was supported by the Fundamental Research Funds for the Central public welfare research institutes (2020YJSZX-3), National Natural Science Foundation of China (No. 82104656) and Beijing Municipal Natural Science Foundation (7214294).

## Conflict of Interest

The authors declare that the research was conducted in the absence of any commercial or financial relationships that could be construed as a potential conflict of interest.

## Publisher's Note

All claims expressed in this article are solely those of the authors and do not necessarily represent those of their affiliated organizations, or those of the publisher, the editors and the reviewers. Any product that may be evaluated in this article, or claim that may be made by its manufacturer, is not guaranteed or endorsed by the publisher.
