# Corrigendum: Use of Anti-angiogenic Drugs Potentially Associated With an Increase on Serum AST, LDH, CK, and CK-MB Activities in Patients With Cancer: A Retrospective Study

**DOI:** 10.3389/fcvm.2021.828274

**Published:** 2022-01-06

**Authors:** Qi Zheng, Hanzhou Wang, Wei Hou, Ying Zhang

**Affiliations:** ^1^Department of Pneumology, Guang'anmen Hospital, China Academy of Chinese Medical Sciences, Beijing, China; ^2^Department of Oncology, Guang'anmen Hospital, China Academy of Chinese Medical Sciences, Beijing, China

**Keywords:** anti-angiogenic drugs, cancer, AST, LDH, CK, CK-MB

In the published article, there was an error regarding the affiliation for Qi Zheng, Wei Hou and Ying Zhang. As well as having affiliation 1, they should have “Department of Oncology, Guang'anmen Hospital, China Academy of Chinese Medical Sciences, Beijing, China.”

The authors apologize for this error and state that this does not change the scientific conclusions of the article in any way. The original article has been updated.

## Publisher's Note

All claims expressed in this article are solely those of the authors and do not necessarily represent those of their affiliated organizations, or those of the publisher, the editors and the reviewers. Any product that may be evaluated in this article, or claim that may be made by its manufacturer, is not guaranteed or endorsed by the publisher.

